# Impact of Internally Developed Electronic Prescription on Prescribing Errors at Discharge from the Emergency Department

**DOI:** 10.5811/westjem.2017.6.32037

**Published:** 2017-07-14

**Authors:** Eveline Hitti, Hani Tamim, Rinad Bakhti, Dina Zebian, Afif Mufarrij

**Affiliations:** * American University of Beirut Medical Center, Department of Emergency Medicine, Beirut, Lebanon; † American University of Beirut Medical Center, Department of Internal Medicine and Clinical Research Institute, Beirut, Lebanon

## Abstract

**Introduction:**

Medication errors are common, with studies reporting at least one error per patient encounter. At hospital discharge, medication errors vary from 15%–38%. However, studies assessing the effect of an internally developed electronic (E)-prescription system at discharge from an emergency department (ED) are comparatively minimal. Additionally, commercially available electronic solutions are cost-prohibitive in many resource-limited settings. We assessed the impact of introducing an internally developed, low-cost E-prescription system, with a list of commonly prescribed medications, on prescription error rates at discharge from the ED, compared to handwritten prescriptions.

**Methods:**

We conducted a pre- and post-intervention study comparing error rates in a randomly selected sample of discharge prescriptions (handwritten versus electronic) five months pre and four months post the introduction of the E-prescription. The internally developed, E-prescription system included a list of 166 commonly prescribed medications with the generic name, strength, dose, frequency and duration. We included a total of 2,883 prescriptions in this study: 1,475 in the pre-intervention phase were handwritten (HW) and 1,408 in the post-intervention phase were electronic. We calculated rates of 14 different errors and compared them between the pre- and post-intervention period.

**Results:**

Overall, E-prescriptions included fewer prescription errors as compared to HW-prescriptions. Specifically, E-prescriptions reduced missing dose (11.3% to 4.3%, p <0.0001), missing frequency (3.5% to 2.2%, p=0.04), missing strength errors (32.4% to 10.2%, p <0.0001) and legibility (0.7% to 0.2%, p=0.005). E-prescriptions, however, were associated with a significant increase in duplication errors, specifically with home medication (1.7% to 3%, p=0.02).

**Conclusion:**

A basic, internally developed E-prescription system, featuring commonly used medications, effectively reduced medication errors in a low-resource setting where the costs of sophisticated commercial electronic solutions are prohibitive.

## INTRODUCTION

Medication errors frequently result in adverse drug events. These errors greatly impact patient safety, representing the leading cause for injuries and death.^1^ Studies have reported at least one error per patient encounter.^2^ An emergency department (ED) setting is believed to be particularly sensitive to medication errors due to exposure to new patients, time constraints, frequent interruptions and limited patient history.^1^, ^3^ Additionally, there is a higher frequency of prescriptions in this setting, with more than 75% of ED visits resulting in drug administration or prescription dispensing.^4^ Errors at discharge in particular are also common, varying from 15%–38%.^5^, ^6^, ^7^, ^8^ Of discharged patients from the hospital, 23% encountered at least one adverse event and 72% of the adverse events were attributed to medications errors.^9^

To our knowledge, a total of two studies have looked at the impact of electronic (E)-prescription error rates at discharge from the ED. Bizovzi et al. found that a commercial E-prescription system was three times less likely to result in errors and five times less likely to demand pharmacist clarification than hand-written (HW) prescriptions within the ED.^10^ A similar effect was reported at discharge in a pediatrics ED with a commercially-based system.^11^

This study examined the effect of introducing a low-cost, internally developed E-prescription system with a list of commonly prescribed medications to the ED at a tertiary care center in Lebanon, on prescription errors compared to HW-prescriptions.

## METHODS

### Study Setting

This study was conducted at the ED of the American University of Beirut Medical Center, the largest tertiary care center in Lebanon, with around 49,000 patient visits per year. The ED is staffed by attendings around the clock along with residents from multiple different services for adult patients (internal medicine, family medicine, surgery and obstetrics residents) and pediatric patients (family medicine and pediatrics residents). The majority of our patients are covered by private third-party payers (67%), while the remaining pay out of pocket. The ED uses an internally developed dashboard system that allows for patient tracking, electronic diagnostics ordering and review of prior visits and diagnostics results. All ED medication ordering throughout the ED stay is done through hand-written orders (HW), including at discharge.

### Study Design

We conducted a pre- and post-intervention study with a random sample of patients selected from the pre- and postintervention period. The pre-intervention phase, which included the HW-prescription at discharge, ran from November 1, 2010–June 30, 2011, while the post-intervention phase, which included the E-prescriptions, ran from November 1, 2011–June 30, 2012. These periods were selected to allow for a wash-out period, specifically one month pre-introduction of the E-prescription and two months post-introduction, during which piloting and implementation was occurring. Approval for this study was granted by our institutional review board.

### Sample selection

Patients eligible in this study were of all ages, genders, and diagnoses, with at least one prescription at discharge, either HW or electronic. We excluded patients whose charts were not scanned into the electronic medical record or if the discharge prescription was missing. We randomly selected charts for the pre-intervention month, by selecting every 10^th^ admission medical record number, checking for the presence of a discharge prescription. If so, the patient was included in the study. This process was repeated until the target number of patients was reached. We also used this method for the postintervention group.

Population Health Research Capsule

What do we already know about this issue?

Commercially available electronic prescription systems decrease prescription errors at ED discharge however they are cost-prohibitive in resource limited settings.

What was the research question?

Assess the impact of introducing an internally developed, low-cost electronic prescription system on prescription error rates at ED discharge.

What was the major finding of the study?

An electronic prescription system featuring commonly used ED medications reduced prescription errors at ED discharge.

How does this improve population health?

Reducing prescription errors at discharge from the ED, by applying a basic electronic prescription system, can prevent adverse drug events and improve quality of care.

### Power calculation

Although the HW-prescribing error rate in the literature ranges between 15–46%,^12^, ^13^ for the sample size calculation of the current study, we considered a rate of 50%, since it yields the highest sample size (most conservative). Accordingly, we estimated that a sample size of 770 patients in each group was needed to detect a 7% reduction in error rates postintervention, with an 80% power and an alpha level of 5%, assuming one discharge prescription per patient.

### Intervention

An electronic discharge process was internally developed by a team that included an emergency-physician champion working with the hospital information technology (IT) team and director of pharmacy. The electronic discharge module was introduced on August 1, 2011. The new system included forced fields for diagnoses, an optional section for follow-up care, optional patient education handouts and a prescription section that included 166 commonly prescribed medications with the generic name of the medication, strength, dose, frequency, route, and duration. The list was developed based on historical data of commonly prescribed medications from the ED, in addition to faculty input. When deciding on common medication categories where multiple options exist, we included the ones on hospital formulary, e.g., esomeprazole rather than pantoprazole. For pediatrics, the list included the medication, strength and recommended dosing only on a mg/kg basis, where the final dose required manual calculation. Hospital pharmacy reviewed the final list for accuracy and availability of medications in the local market. The system did not include allergy- or medication-reconciliation functions. Physicians could also free text additional medications without forced fields. The time to complete and print the E-prescription was around 30 seconds. The total cost of development and implementation including IT personnel time, ED medical director time and pharmacist time was approximately $4,300 U.S. in our setting.

### Data collection

The methods followed in this study adhere to the criteria suggested by Worster et al. for retrospective chart review.^14^ We used a data collection sheet to facilitate extracting the information and to de-identify the phase of the study. Two research assistants who were trained prior to data collection and monitored throughout transcribed both the HW- and E-prescriptions into a Microsoft Excel database. We reviewed medical charts retrospectively to collect patient-specific demographic and medical data including age, gender, emergency severity index (ESI), discharge diagnosis, allergies, home and discharge medications (number and all prescription-related information on medication name, dose, strength, frequency, route, and duration) and number of handovers as reflected by attending shift changes during the patient’s stay.

Moreover, we used an administrative database to collect workload and scheduling metrics that might affect error rates. These included ED visit volume per day, weekday/weekend shift, shift type (morning shift, which ran from 8am–4pm; evening shift, 4pm to midnight; and night shift, midnight to 8am).

### Definitions and identification of errors

The definition of errors in each prescription was according to the error list provided in Table 1. Duplication with discharge medication was considered an error when two medications of the same family were included in the discharge prescription, for example, ibuprofen and naproxen. We considered duplication with home medications an error when at least one of the discharge medications was of the same family as one of the home medications and there were no instructions to hold or stop the home medication. Drugs were reviewed for interactions with all the medications listed in the discharge prescription list and the home medication list. We used Lexicomp® drug interaction software to check for all interactions and risk ratings as per the software, where risk A involved no known interaction, risk B required no action, risk C required monitoring therapy, risk D required consideration of therapy modification and risk X required avoidance of combination.^15^ All risk D and X interactions were considered an error.

We included drug allergy error if the patient was discharged on a medication that was listed as an allergy in the patient record, or was of the same family of the allergy medication. Lexicomp software was also used to review all medication dosing, frequency, and duration recommendations. A prescription was considered to have an error in these categories if there was deviation from the Lexicomp recommendation. Incorrect strength was considered an error if the strength of the medication was not one available in the local market per the Lebanese Ministry of Public Health formulary list.^16^ A medication was considered illegible if the research assistant was unable to read it. The two research assistants who extracted the data completed the error scoring. Moreover, to verify the scoring, a clinical pharmacist, who was blinded to the purpose and phase of the study, reviewed the de-identified data and scored them independently. Finally, any discrepancy between the scoring of the research assistants (RAs) and the pharmacist was resolved by discussion with the principal investigator (PI) of the study, as well as the director of clinical pharmacy at our institution.

### Outcomes and classification of errors

#### Primary outcomes

We classified errors directly impacted by the intervention as primary outcomes. These included incorrect route, dose, or frequency, or strength, illegibility and missing duration, dose, frequency, or strength.

#### Other outcomes

Errors that were not directly targeted by the intervention but were felt to potentially impact patient safety were considered other outcomes. These included the following: duplication with discharge medication, duplication with home medications, interactions of discharge medication with another discharge medication, interaction of discharge medications with home medications and drug/allergy interaction.

### Classifications

A priori, we categorized those under 14 years of age as pediatric, and those above as adults. This classification was based on a previous study, where the age group corresponds to a typical weight of 50kg or less and is likely to need weight-based prescription dosing.^10^ The error types were classified into three groups: incorrect errors (incorrect route, dose, frequency, and strength), missing information errors (missing duration, dose, frequency, and strength) or illegible errors. Error types were also grouped as high or low risk. We considered errors high risk if they had the potential to cause significant harm and were not part of routine pharmacist verification practice. All missing-information errors were considered low risk as pharmacy verification would be required to fill the prescription. High-risk errors included duplication with discharge medication, drug/drug interaction with home medications, drug/drug interaction with discharge medications, drug/allergy interaction, incorrect dose, incorrect frequency, incorrect strength, and duplication with home or discharge medication. Low-risk errors included incorrect route, missing duration, missing dose, missing frequency, and missing strength.

### Statistical Analysis

We used the Statistical Package for Social Sciences (SPSS)® for the data management and analyses. The distribution of the medication errors and the predictors (sociodemographic characteristics, ED scheduling, ED workload and patient medical status) are presented as means ± standard deviations (SD) and frequencies and percentages for the continuous and categorical variables, respectively. We used Pearson’s chi-squared and one-way Student’s t-test to assess the significance of the association between the predictor factors (continuous and categorical) and the medication error.

We performed a multivariate analysis using logistic regression to find the best model that fit the data and explained the association between medication error and all predictor variables, which included the following: type of prescription, age, gender, ESI, number of home medications, number of discharge medications, shift type, ED volume per day and handovers per visit. We conducted a backward selection procedure by fitting medication error with all risk factors found to be significant at the bivariate level, in addition to those considered clinically meaningful. Furthermore, the magnitude of association between the predictor variables and medication errors was determined by calculating the adjusted odds ratios (aOR) and their corresponding 95% confidence intervals (CI). Missing data were not modified, and statistical significance was established at the p-value of 0.05.

## RESULTS

We included a total of 2,883 prescriptions in the study, of which 1,475 (51.2%) were in the pre-intervention period (HW), and 1,408 (48.8%) in the post-intervention (E). Table 2 presents the results of the comparison of the demographic characteristics and the ED workload data between the pre- and post-intervention periods. Overall, characteristics of both patient populations were similar, although there was a slight decrease in the number of home medications and discharge medications per patient in the postintervention period (1.3 prescription per patient compared to 1.1, p=0.002). As for the workload characteristics, the ED workload per day, though not clinically significant, was lower in the postintervention period (132.4 vs 134.1, p=0.002) with more patients presenting during the night shift (31.1% vs 25.2%, p=0.001).

Overall, E-prescriptions were significantly associated with a reduced error rate (67.7% vs 45.5%, p<0.0001) (OR=0.40, 95%, CI [0.34–0.46]) (Table 3). More specifically, E-prescriptions were associated with a significant reduction of “missing dose” errors (11.3% vs. 4.3%, OR=0.36, 95% CI [0.26–0.48], p <0.0001), “missing frequency” errors (3.5% vs. 2.2%, OR=0.63, 95% CI [0.40–0.99], p=0.04), and “missing strength” errors (32.4% vs 10.2%, OR=0.24, 95% CI [0.1–0.29], p <0.0001). “Legibility” also significantly improved with E-prescriptions (0.7% vs 0.1%, OR=0.10, 95% CI [0.01–0.73], p=0.005). On the other hand, E-prescriptions were associated with a significant increase of “incorrect strength” errors (1.5% vs. 3.6%, OR=2.48, 95% CI [1.50–4.12], p <0.0001) and “duplication with home medication” (1.7% vs. 3.0%, OR=1.78, 95% CI [1.08–2.94], p = 0.02).

When classified into broad categories of prescription error types, “missing information” (which includes missing duration, route, dose, strength, name, and frequency) was the most common type of error to occur overall (47.5%) and was significantly less common in E-prescriptions as compared to the HW-prescriptions (35.5% vs 59.0%, respectively, p <0.0001) (Table 4). On the other hand, “incorrect information” (which includes incorrect route, dose, and frequency) errors were more common in E-prescriptions, with borderline statistical significance (8.9% vs 7.0%, p=0.05).

Table 5 presents the comparison between the HW- and E-prescriptions by risk level of errors. Low-risk prescribing errors were the most common type of errors in both groups, yet it was found to be less in the E-prescriptions as compared to the HW (35.5% vs. 59.1%, p <0.0001). Similarly, the illegible errors were less in the E-prescription (0.1% vs 0.7%, p= 0.005). On the other hand, high-risk errors were more common in the E-prescriptions as compared to the HW ones (18.2% vs 15.0%, p = 0.02).

The results of the multivariate logistic regression analysis for the predictors of all types of medication errors are presented in Table 6. After adjusting for potentially confounding factors, it was found that E-prescriptions were a strong predictor of fewer errors (adjusted OR = 0.40, 95% CI [0.35 – 0.47], p<0.0001).

## DISCUSSION

This pre- / post-intervention study demonstrates that the implementation of a low-cost, internally developed E-prescription system, featuring a list of commonly used medications, with no decisional support features, can effectively reduce the number of medication errors. While multiple studies have demonstrated the impact of sophisticated E-prescription system on reducing prescribing errors at discharge, the expense of such systems may be prohibitive in low-resource settings.

The types of errors significantly reduced with E-prescriptions in our study were the following: missing dose, missing frequency, missing strength, and illegibility errors. In terms of broad categories of errors, low-risk errors, illegible errors and missing-information errors emerged as significantly reduced by E-prescription. By contrast, incorrect information errors were more common in E-prescriptions. This was mainly due to an incorrect strength of one commonly used medication that was included in the final list and perpetuated in all the E-prescriptions.

Our study revealed no improvement in the other outcomes. In fact, duplication with home medications increased upon E-prescription use while no such effect was noted for drug-interaction errors and drug-allergy errors. This was likely because the design of the internally developed system in our study did not specifically target high-risk errors or include drug-allergy checking, medication reconciliation, and drug-drug interaction features. Since no controls for these errors were introduced, the difference in corresponding error rates between pre-and post-intervention was expectedly not large. Overall, this is in line with previous studies in which computerized systems were not as effective with high-risk medication errors.^17^, ^18^ Such high-risk errors would require developing more sophisticated programs that include fields for entering home medications and allergies, which could then be crosschecked with the discharge medications for interactions/contraindications.

In addition, although the current system includes a list of commonly prescribed medications, a free-text option remained available to providers. This may have reduced the impact on missing-information errors. Implementing a program that makes some elements mandatory would be an easy, low-cost modification that would further mitigate this type of error.

Features of commercially available E-prescription systems range from basic medication lists to robust decision-making support with medication reconciliation processes. While decision support capability to address high-risk errors is an important component of commercially available E-prescription systems, such complex systems can cost up to $29,000 per physician for the first year and $4,000 annually thereafter.^19^ Even the cost of commercially available E-prescriptions systems with basic features is high, ranging between $1,500 and $4,000 per physician. Such costs are likely unaffordable in low-resource settings where internally developed solutions may offer more feasible options.

## LIMITATIONS

There are a few limitations to this study. Firstly, this intervention was implemented across a single institution, which may limit generalizability. Given the pre- /post study design, some physician- and patient-related characteristics may have varied and introduced a bias into the results. Additionally, the outcome and consequences of medication errors and their severity, including adverse drug events, were not measured and assessed. Moreover, although discrepancy between abstractors was resolved through a systematic process with the PI, nevertheless, inter-observer reliability was not tested.

## CONCLUSION

An E-prescription system that includes a common list of ED medications considerably decreased the frequency of the majority of prescription errors. To date, no studies have investigated the impact of a low-cost electronic, internally developed system in an ED where resources are limited and acquiring comprehensive and commercial E-solutions is cost-prohibitive. The developed system is comparatively more basic than currently available systems and uses entirely internal resources. The decrease in error rates introduced by this cost-effective system supports its implementation, particularly in developing countries with limited financial resources.

## Figures and Tables

**Table 1:** Types of errors in prescriptions for discharge medication, and corresponding risk level.

Description	Risk level classification
High-risk errors	
Duplication with discharge medication	High
Duplication with home medication	High
Drug/drug interaction (D/H)	High (type D and X)
Drug/drug interaction (D/D)	High (type D and X)
Drug/allergy interaction	High
Incorrect dose	High
Incorrect frequency	High
Incorrect strength of drug	High
Low-risk errors	
Incorrect route	Low
Missing duration	Low
Missing dose	Low
Missing frequency	Low
Missing strength of drug	Low
Illegibility	Illegible

Drug/drug interaction (D/H): interaction of discharge medications with home medications. Drug/drug interaction (D/D): interactions of discharge medication with another discharge medication. Type D required consideration of therapy modification and type X required avoidance of combination.

**Table 2:** Association between the demographic variables and the use of handwritten (HW) or electronic (E) prescription.

		Pre-intervention HW number (%)	Post-intervention E number (%)	
				
Total sample		N=1475	N=1408	p value
Patient characteristics				
Age (years)	(Mean, ±SD)	31.4 (±20.9)	31.3 (±20.0)	0.81
Male gender		746 (50.6%)	715 (50.8%)	0.91
ESI	(Mean, ±SD)	3.3 (±0.6)	3.3 (±0.7)	0.10
Pediatric patients	Pediatric	320 (21.7%)	268 (19.0%)	0.08
Number of home medications/patient	(Mean, ±SD)	1.3 (±1.7)	1.1 (±1.6)	0.002
Number of discharge medications/patient	(Mean, ±SD)	2.4 (±1.0)	2.3 (±1.0)	0.001
ED workload				
Shift				0.001
Morning		528 (35.8%)	485 (34.4%)	
Evening		575 (39.0%)	485 (34.4%)	
Night		372 (25.2%)	438 (31.1%)	
Handovers per visit	(Mean, ±SD)	1.1 (±0.3)	1.2 (±0.4)	0.33
ED volume per day	(Mean, ±SD)	134.1 (±13.4)	132.4 (±16.4)	0.002

*HW,* handwritten prescriptions*; E,* electronic prescriptions; *ESI*, Emergency Severity Index; *SD*, standard deviation.

**Table 3:** Association between the type of errors and the use of handwritten (HW) or electronic (E) prescriptions.

	Pre-intervention HW number (%)	Post-intervention E number (%)		
				
Total sample	N=1475	N=1408	Crude OR (95% CI)	p value
All type errors	999 (67.7%)	641 (45.5%)	0.40 (0.34 – 0.46)	<0.0001
Duplication with discharge medication	5 (0.3%)	4 (0.3%)	0.84 (0.22 – 3.13)	1.00
Drug/drug interaction (D/H)	107 (7.3%)	96 (6.8%)	0.94 (0.70 – 1.25)	0.65
Drug/drug interaction (D/D)	51 (3.5%)	55 (3.9%)	1.14 (0.77 – 1.67)	0.52
Drug/allergy interaction	0 (0.0%)	2 (0.1%)	-	0.24
Incorrect drug	2 (0.1%)	1 (0.1%)	0.52 (0.05 – 5.78)	1.00
Incorrect dose	40 (2.7%)	26 (1.8%)	0.68 (0.41 – 1.11)	0.12
Incorrect frequency	51 (3.5%)	57 (4.0%)	1.18 (0.80 – 1.73)	0.40
Illegibility	11 (0.7%)	1 (0.1%)	0.10 (0.01 – 0.73)	0.005
Missing duration	398 (27.0%)	410 (29.1%)	1.11 (0.95 – 1.31)	0.20
Missing dose	166 (11.3%)	61 (4.3%)	0.36 (0.26 – 0.48)	<0.0001
Missing frequency	51 (3.5%)	31 (2.2%)	0.63 (0.40 – 0.99)	0.04
Missing strength	478 (32.4%)	144 (10.2%)	0.24 (0.19 – 0.29)	<0.0001
Incorrect strength	22 (1.5%)	51 (3.6%)	2.48 (1.50 – 4.12)	<0.0001
Duplication with home medication	25 (1.7%)	42 (3.0%)	1.78 (1.08 – 2.94)	0.02

*HW*, handwritten prescriptions; *E*, electronic prescriptions.

Drug/drug interaction (D/H): interaction of discharge medications with home medications. Drug/drug interaction (D/D): interactions of discharge medication with another discharge medication.

**Table 4:** Association between the types of prescribing errors by broad categories and the use of electronic or handwritten prescription

	Pre-intervention HW number (%)	Post-intervention E number (%)	
			
Total sample	N=1475	N=1408	p value
Drug interaction errors	128 (8.7%)	140 (9.9%)	0.24
Incorrect information errors	103 (7.0%)	126 (8.9%)	0.05
Illegible errors	11 (0.7%)	1 (0.1%)	0.005
Missing information errors	870 (59.0%)	500 (35.5%)	<0.0001
Drug allergy errors	0 (0.0%)	2 (0.1%)	0.24

*HW*, handwritten prescriptions, *E*, electronic prescriptions

**Table 5:** Comparison between handwritten and electronic prescriptions according to the risk level.

	Pre-intervention HW number (%)	Post-intervention E number (%)	
			
Total sample	N=1475	N=1408	p value
All errors	985 (66.8%)	626 (44.5%)	<0.0001
Low-risk errors	871 (59.1%)	500 (35.5%)	<0.0001
High-risk errors	221 (15.0%)	256 (18.2%)	0.02
Illegible errors	11 (0.7%)	1 (0.1%)	0.005

*HW*, handwritten prescriptions, *E*, electronic prescriptions

**Table 6:** Multivariate analysis for the predictors of all types of medication errors vs no errors (hierarchical method imposing the type of prescription).

Predictors	Adjusted OR (95%CI)	P value
Type of prescription (handwritten/electronic)	0.40 (0.35 – 0.47)	<0.0001
Age	1.01 (1.01 – 1.02)	<0.0001
Pediatrics	1.38 (1.06 – 1.78)	0.02
Number of home medications per patient	1.18 (1.11 – 1.25)	<0.0001

Variables entered in the model include the following: type of prescription, total visits, ED volume, age, gender, (Emergency Severity Index), pediatric (as compared to adult) patients, number of home medications per patient, number of discharge medications per patient, shift evening, shift night.
